# The Stress and Anxiety to Viral Epidemics-6 Items (SAVE-6) Scale: A New Instrument for Assessing the Anxiety Response of General Population to the Viral Epidemic During the COVID-19 Pandemic

**DOI:** 10.3389/fpsyg.2021.669606

**Published:** 2021-06-04

**Authors:** Seockhoon Chung, Myung Hee Ahn, Sangha Lee, Solbi Kang, Sooyeon Suh, Yong-Wook Shin

**Affiliations:** ^1^Department of Psychiatry, ASAN Medical Center, University of Ulsan College of Medicine, Seoul, South Korea; ^2^Division of Psychiatry, Health Screening and Promotion Center, ASAN Medical Center, Seoul, South Korea; ^3^Department of Psychiatry, Ajou University School of Medicine, Suwon, South Korea; ^4^Department of Psychology, Sungshin Women’s University, Seoul, South Korea

**Keywords:** stress, anxiety, mental health, general population, COVID-19

## Abstract

The general population has reported experiencing anxiety due to the COVID-19 pandemic. This study explored the validity and utility of the Stress and Anxiety to Viral Epidemics-6 items (SAVE-6) scale for measuring the anxiety response of the general population to the viral epidemic. About 1,009 respondents participated in an online survey. Of these, 501 (49.7%) participants were rated as having at least a mild degree of anxiety response to the viral epidemic (SAVE-6 score ≥ 15), while 90 (8.9%) and 69 (6.8%) participants were rated as having moderate degree of depression and anxiety, respectively. The SAVE-6 scale showed a good internal consistency (Cronbach’s alpha = 0.815). Parallel analysis suggested a one-factor structure for the measure. The SAVE-6 scale was found to be a reliable, valid, and useful brief measure that can be applied to the general population.

## Introduction

The COVID-19 pandemic has disrupted the daily lives of many individuals, with them experiencing various psychiatric issues, including depression, anxiety, insomnia, and post-traumatic stress. Particularly, people experience the fear of infection, both for themselves and their loved ones, or of spreading the infection to others. The prevalence of anxiety symptoms in the pandemic era reported ranging from 6.33 to 50.9% (Xiong et al., 2020). Studies have assessed the anxiety levels of individuals using various rating scales, such as the Generalized Anxiety Disorder-seven items (GAD-7; Hou et al., 2021), Zung’s Self-rating Anxiety Scale (SAS; Ran et al., 2020), and the Depression Anxiety and Stress Scale (Wang et al., 2020). However, these scales do not specifically assess anxiety dealing with the COVID-19 pandemic. Therefore, a rating scale specific to the viral epidemic needs to be developed to determine the actual effects of the COVID-19 pandemic on the anxiety of an individual.

Several rating scales were developed and applied in 2020 in response to the pandemic: the five-item Coronavirus Anxiety Scale developed by Lee (2020a), the COVID-19-Anxiety Questionnaire modified by Petzold et al. (2020) based on the DSM-5 Severity Measure for Specific Phobia-Adult Scale, the seven-item Fear of COVID-19 Scale developed by Ahorsu et al. (2020), the four-item Obsession with COVID-19 Scale developed by Lee (2020b), the 11-item Coronavirus Pandemic Anxiety Scale developed by Bernardo et al. (2020), the two-factor nine-item COVID-19 Anxiety Syndrome Scale (six items for perseverance and three items for avoidance) developed by Nikcevic and Spada (2020), and the seven-item COVID-19 Anxiety Scale developed by Silva et al. (2020). These scales inquired about the anxiety of, repetitive thoughts of, or anxiety-related physiological arousal symptoms in an individual. Originally, we developed the Stress and Anxiety to Viral Epidemics-9 (SAVE-9) items scale specifically for healthcare workers. It consisted of items inquiring about apprehension or thoughts of an individual about the risk of infection, about the consequent influence on their physical health, or about avoidance of others (Chung et al., 2020). The SAVE-9 scale was designed to have two factors: first, anxiety about the viral epidemic factor, including six items (SAVE-6) and second, work-related stress associated with the viral epidemic, including three items (SAVE-3). We previously validated the SAVE-9 questionnaire and verified its utility among healthcare workers. In this study, we hypothesized that the SAVE-6 scale can be used for measuring anxiety in response to the viral epidemic among the general population. We also explored the psychometric properties of the SAVE-6 scale and determined the appropriate cut-off point of the scale with respect to the general anxiety symptoms.

## Materials and Methods

### Participants and Procedure

This study was conducted *via* an anonymous online survey system through EMBRAIN, a professional research company.^1^ The survey collected 1,009 responses from January 14 to 20, 2021. The participants voluntarily responded to the survey. The mean age of the participants was 44.3 (±13.5) years, with 51% (*n* = 515) male population. The study protocol was approved by the Institutional Review Board of Sungshin Women’s University, Seoul, South Korea (SSWUIRB-2020-040). Written informed consent was waived.

### Assessment of Symptoms

#### SAVE-6

The SAVE-6 scale is a subcategory of the SAVE-9 scale^2^ developed originally for measuring stress and anxiety due to the viral epidemic among healthcare workers (Chung et al., 2020). Each of the six items is rated on a five-point Likert scale ranging from 0 (never) to 4 (always). The cut-off score of the SAVE-6 scale has been reported to be 15, equivalent to at least a mild degree or ≥ 5 on the GAD-7 scale. The total score on the SAVE-6 scale ranges from 0 to 24, with higher scores reflecting higher levels of anxiety response to the viral epidemic.

#### GAD-7

The GAD-7 scale is a self-report questionnaire for measuring general anxiety (Spitzer et al., 2006). Each item is scored on a four-point Likert scale (0 = not at all to 3 = nearly every day). Scores range from 0 to 21, with higher scores reflecting higher levels of anxiety. The cut-off points for anxiety are 0–4 (minimal), 5–9 (mild anxiety), 10–14 (moderate), and 15–21 (severe).

#### Patient Health Questionnaire-9

The PHQ-9 scale is a self-report questionnaire for measuring depression (Kroenke et al., 2001). Each item is rated on a four-point Likert scale (0 = not at all to 3 = nearly every day). Scores range from 0 to 27, with higher scores reflecting severe depression. The cut-off points for depression are 0–4 (minimal), 5–9 (mild), 10–14 (moderate), 15–19 (moderately severe), and 20–27 (severe).

### Statistical Analyses

We conducted an independent *t*-test and the chi-square test to examine the gender differences in clinical variables or rating scale scores using the IBM SPSS Statistics for Windows, version 21.0. We also performed Spearman’s correlation to examine the association of scores from the SAVE-6 scale with demographic variables and rating scale scores since the distribution of PHQ-9 and GAD-7 scores were not within the normal limit. We hypothesized a one-factor model for the SAVE-6 scale based on the previous analysis on healthcare workers (Chung et al., 2020). The normality assumption was checked by using skewness and kurtosis for an acceptable limit of range ±2 (Gravetter and Wallnau, 2014). After examining the Kaiser-Meyer-Olkin (KMO) measure of sampling adequacy and Bartlett’s test of sphericity to explore the data suitability, exploratory factor analysis (EFA) was conducted to evaluate the construct validity. In EFA, we used the principal axis factor (PAF) extraction method with a Pearson’s correlation matrix and promax rotation. To determine the number of factors to be retained, the scree test and the parallel analysis test (Horn, 1965; Glorfeld, 1995; Timmerman and Lorenzo-Seva, 2011), based on minimum rank factor analysis (MRFA; Lorenzo-Seva and Ferrando, 2006), with a 95-percentile threshold, based on the polychoric correlation matrix, were conducted using FACTOR, version 10.10.03 (Lorenzo-Seva and Ferrando, 2006) program. The reliability and internal consistency of the factor were examined using Cronbach’s alpha and McDonald’s omega coefficient to verify the dimensionality of the SAVE-6 scale. Finally, the receiver operating characteristic (ROC) analysis was performed to explore the appropriate cut-off score of the SAVE-6 scale in accordance with generalized anxiety symptoms.

## Results

### Demographic Characteristics

Table 1 presents the demographic characteristics of the patients. There is no significant gender difference in clinical variables and rating scale scores except in the SAVE-9 scale score. Among the sample, 90 (8.9%) and 69 (6.8%) participants scored above the cut-off for clinical depression symptoms (PHQ-9 ≥ 10) and generalized anxiety (GAD-7 ≥ 10), respectively. Among the respondents, 222 (20.0%) reported knowing a person that had been infected, 80 (7.9%) reported having the experience of being quarantined, 6 (0.6%) reported the experience of being infected themselves, and 186 (18.4%) reported having a serious medical illness.

**Table 1:** Demographic characteristics of participants (*N* = 1,009).

Variables	Male (*N* = 515)	Female (*N* = 494)	*p*-value
	Mean ± *SD*, *N* (%)		
Age (Years)	44.0 ± 13.5	44.7 ± 13.5	0.59
19~29 years old	99 (19.2%)	89 (18.0%)	0.94
30~39 years old	98 (19.0%)	89 (18.0%)	
40~49 years old	114 (22.1%)	109 (22.1%)	
50~59 years old	118 (22.9%)	116 (23.5%)	
60~69 years old	86 (16.7%)	91 (18.4%)	
Marital status (Single)	173 (33.6%)	142 (28.7%)	0.10
Education			
High school and under	121 (23.5%)	134 (27.1%)	0.27
University or college	331 (64.3%)	311 (63.0%)	
Postgraduate	63 (12.2%)	49 (9.9%)	
Region			
Metropolitan Cities (Seoul, Busan, Daegu, Incheon, Gwangju, Daejeon, Ulsan)	222 (43.1%)	293 (56.9%)	0.38
Suburban Provinces (Gyeonggi, Gangwon, Chungcheong, Jeolla, Gyeongsang, Jeju)	227 (46.0%)	267 (54.0%)	
COVID-19 questions			
Is there anyone you know who has been infected with COVID-19? (Yes)	123 (23.9%)	99 (20.0%)	0.15
Did you experience being quarantined for having been infected with COVID-19? (Yes)	45 (8.7%)	35 (7.1%)	0.35
Did you experience being infected with COVID-19? (Yes)	4 (0.8%)	2 (0.4%)	0.69
Do you have any serious medical problems, such as cardiovascular or pulmonary disease? (Yes)	91 (17.7%)	95 (19.2%)	0.57
Rating scales			
Stress and Anxiety to Viral Epidemics-6 (SAVE-6)	14.0 ± 4.7	14.7 ± 4.6	0.02
Patient Health Questionnaire-9 (PHQ-9)	6.0 ± 5.0	6.4 ± 4.9	0.28
Depression (PHQ-9 ≥ 10)	44 (8.5%)	46 (9.3%)	0.74
Generalized Anxiety Disorder-7 (GAD-7)	3.2 ± 3.9	3.4 ± 3.7	0.53
Generalized anxiety (GAD-7 ≥ 10)	36 (7.0%)	33 (6.7%)	0.90

The SAVE-6 scores were significantly higher among respondents who were rated as having depression [PHQ-9 ≥ 10, *t* (1,007) = 9.29, and *p* < 0.001] and generalized anxiety [GAD-7 ≥ 10, *t* (1,007) = 8.34, and *p* < 0.001]. Moreover, the SAVE-6 scale scores were significantly higher among women [*t* (1,007) = 2.38 and *p* = 0.018] when compared with men, among people with a serious disease [*t* (1,007) = 2.11 and *p* = 0.035], and among people who knew a person infected with COVID-19 (*t* (1,007) = 2.07 and *p* = 0.038). However, no significant differences were observed with respect to the area of residence (*p* = 0.19), to the experience of being infected (*p* = 0.55), and to the experience of being quarantined (*p* = 0.09).

### Factor Structure of the SAVE-6 Scale

The normality assumption was checked. It revealed that the distribution of each of the six items was within the normal limit (Table 2). The KMO measure (0.82) and the Bartlett’s test of sphericity (*p* < 0.001) showed adequacy for running EFA. The EFA with PAF extraction, the polychoric correlation, and the promax rotation suggested a one-factor model of the SAVE-6 scale based on the Kaiser Criterion method with an eigenvalue above 1.00 (eigenvalue = 2.635, 42.3% of the variance).

**Table 2:** Frequencies of answers of participants to each of the SAVE-6 item.

Items	Response scale, *N* (%)					Descriptive	Skewness	Kurtosis	Factor loading
	Never	Rarely	Sometimes	Often	Always	Mean ± *SD*			
1. Are you afraid the virus outbreak will continue indefinitely?	12 (1.2%)	51 (5.1%)	132 (13.1%)	473 (46.9%)	341 (33.8%)	3.07 ± 0.88	−1.008	1.047	0.666
2. Are you afraid your health will worsen because of the virus?	30 (3.0%)	101 (10.0%)	216 (21.4%)	427 (42.3%)	235 (23.3%)	2.73 ± 1.02	−0.679	−0.033	0.844
3. Are you worried that you might get infected?	55 (5.5%)	132 (13.1%)	262 (26.0%)	397 (39.3%)	163 (16.2%)	2.48 ± 1.08	−0.523	−0.332	0.807
4. Are you more sensitive toward minor physical symptoms than usual?	72 (7.1%)	180 (17.8%)	259 (25.7%)	367 (36.4%)	131 (13.0%)	2.30 ± 1.12	−0.366	−0.662	0.679
5. Are you worried that others might avoid you even after the infection risk has been minimized?	281 (27.8%)	368 (36.5%)	156 (15.5%)	133 (13.2%)	71 (7.0%)	1.35 ± 1.21	0.695	−0.501	0.593
6. Do you worry your family or friends may become infected because of you?	79 (7.8%)	142 (14.1%)	236 (23.4%)	373 (37.0%)	179 (17.7%)	2.43 ± 1.168	−0.505	−0.562	0.749

Cronbach’s Alpha is 0.815 for total SAVE-6 measure, SD = Standard Deviation.

The scree test and parallel analysis using the MRFA extraction and polychoric correlation were used to identify the adequate number of factors for the scale. We compared the explained real-data eigenvalues with the 95th percentile of random eigenvalues and made a decision where the real-data eigenvalues exceeded the 95th percentile of random eigenvalues. The results suggested that the single-factor structure (real-data eigenvalue = 69.99, 95th percentile of random eigenvalue = 45.40) of the SAVE-6 scale similar to that of the previous study (Chung et al., 2020).

### Reliability of the Scores and Evidence Based on Relations to Other Variables

The SAVE-6 scale showed a good internal consistency reliability (McDonald’s ϖ = 0.818 and Cronbach’s *α* = 0.815). In this sample, the Cronbach’s α of PHQ-9 and GAD-7 were 0.869 and 0.929, respectively. The high scores of SAVE-6 scale scores were significantly correlated with PHQ-9 scores (*ρ* = 0.37, *p* < 0.001) and GAD-7 scores (*ρ* = 0.37, *p* < 0.001). In this study, the ROC analysis revealed that the 15 point of the SAVE-6 scale is appropriate (area under the curve, AUC = 0.706, sensitivity = 70.7%, and specificity = 60.0%) for at least a mild degree of GAD-7 score (≥5), and almost half of the 1,009 respondents (*n* = 501, 49.7%) scored ≥ 15 on the SAVE-6 scale. We also observed that the 17 point of the SAVE-6 scale is in accordance with the moderate degree of GAD-7 (≥10, AUC = 0.768, sensitivity = 72.5%, and specificity = 71.3) and 320 (31.7%) respondents were scored ≥ 17 on the SAVE-6 scale.

## Discussion

We originally developed the SAVE-9 scale for healthcare workers during the pandemic (Chung et al., 2020). We previously found that the SAVE-9 scale could be clustered into two factors: anxiety about the viral epidemic (six items, SAVE-6) and work-related stress associated with the viral epidemic (three items, SAVE-3). In the present study, we investigated the utility of the six-item factor when applied to the general population, labeled as the SAVE-6 scale. We observed that EFA supported a one-factor model of the SAVE-6 scale, consistent with the result of the parallel analysis. The SAVE-6 scale showed good internal consistency reliability. In addition, the ROC analysis revealed that the 15 point of the SAVE-6 scale is appropriate for at least a mild degree of GAD-7 score (≥5).

The SAVE-6 scale was extracted from the original SAVE-9 scale for measuring the behavior or thoughts of healthcare workers during the COVID-19 pandemic. Previous rating scales were developed to inquire about physiological arousal symptoms of individuals associated with clinically elevated fear and anxiety (the Coronavirus Anxiety Scale, Lee, 2020a); feelings of anxiety, nervousness, muscle tension, and behaviors of avoidance (the COVID-19-Anxiety Questionnaire, Petzold et al., 2020); worry, increased heartbeat, or repetitive thoughts (the Fear of COVID-19 scale, Ahorsu et al., 2020; the Coronavirus Pandemic Anxiety Scale, Bernardo et al., 2020; the COVID-19 Anxiety Scale, Silva et al., 2020); or behaviors of avoidance, checking, and worrying (the COVID-19 Anxiety Syndrome Scale, Nikcevic and Spada, 2020). The SAVE-9 scale consists of items inquiring about the apprehension of an individual during the current pandemic situation, work-related stress of healthcare workers, worry about avoidance behavior of others, and concern about their own health and the health of their family members.

Though the results of this study showed a good single model of the SAVE-6 scale with good reliability, we observed a gender difference in the scores of SAVE-6 scale. In this pandemic era, the level of stress or anxiety due to the viral epidemic was reported to be higher among women compared to men in the general population (Hou et al., 2020; Mohammadpour et al., 2020), and even in the special population, such as healthcare workers (Huang et al., 2021; Lee et al., 2021). Silva et al. (2020) also observed the higher level of anxiety among female participants while developing their new rating scale, the COVID-19 anxiety scale. Female preponderance in the anxiety level needs to be considered while developing an anxiety scale targeting the viral epidemic may be expected. Moreover, female preponderance in the anxiety level needs to be considered while developing an anxiety scale targeting the viral epidemic.

The SAVE-9 scale for healthcare workers was originally developed to be brief and practical and to identify individuals who need psychological support. The appropriate cut-off score of the SAVE-9 scale was defined in accordance with at least a mild degree of GAD-7 score to screen healthcare workers who may be vulnerable to COVID-19 infection and consequent work-related stress (Chung et al., 2020). In the previous study, the appropriate cut-off score of factor I of the SAVE-9 scale was defined as point 15 (AUC = 0.728, sensitivity = 0.72, and specificity = 0.61) among healthcare workers. In parallel with the current study, we also observed point 15 of the SAVE-6 scale as a cut-off among the general population (AUC = 0.706, sensitivity = 70.7%, and specificity = 60.0%). In the current study, 49.7% of the participants were rated as having at least a mild degree of anxiety to the viral epidemic using the SAVE-6 scale, while 31.4% of participants were rated as having a mild degree of anxiety with a GAD-7 score ≥ 5. Although the data were not shown in the results, an additional 27.5% of the participants were screened using the SAVE-6 scale among those who were not rated as having anxiety (GAD-7 < 5).

This study had several limitations. First, we did not measure test-retest reliability. Therefore, it was difficult to state the stability of the measure. Second, we could not gather information concerning the employment of the participants. Given that people from certain professions, such as healthcare workers, government officials, and school teachers, are at a higher risk of infection in this pandemic era, the analysis could have benefited from considering the jobs or workplaces of the participants. Last, the results of this study should be interpreted with caution as it is a cross-sectional study. Further studies are needed to generate more information about the general population.

In conclusion, we observed that the SAVE-6 scale is a reliable, valid, and useful brief measure. Future studies should explore the utility of the SAVE-6 scale among the general population using a more representative sample.

## Data Availability Statement

The raw data supporting the conclusions of this article will be made available by the authors, without undue reservation.

## Ethics Statement

The studies involving human participants were reviewed and approved by Institutional Review Board of Sungshin Women’s University, No. SSWUIRB-2020-040. Written informed consent was waived.

## Author Contributions

SC, MA, Y-WS, and SS conceived the study. SS obtained ethics approval. SK, SS, and SL recruited participants and obtained data. SC organized the database and performed statistical analyses. All authors contributed to the article and approved the submitted version.

### Conflict of Interest

The authors declare that the research was conducted in the absence of any commercial or financial relationships that could be construed as a potential conflict of interest.

## Footnotes

^1^www.embrain.com

^2^www.save-viralepidemic.net
